# Axonal injury in asymptomatic individuals preceding onset of multiple sclerosis

**DOI:** 10.1002/acn3.51568

**Published:** 2022-05-03

**Authors:** Daniel Jons, Henrik Zetterberg, Martin Biström, Lucia Alonso‐Magdalena, Martin Gunnarsson, Magnus Vrethem, Kaj Blennow, Staffan Nilsson, Peter Sundström, Oluf Andersen

**Affiliations:** ^1^ Department of Clinical Neuroscience, Institute of Neuroscience and Physiology, The Sahlgrenska Academy University of Gothenburg Gothenburg Sweden; ^2^ Department of Psychiatry and Neurochemistry, Institute of Neuroscience and Physiology, The Dahlgren's Academy University of Gothenburg Gothenburg Sweden; ^3^ Clinical Neurochemistry Laboratory Sahlgrenska University Hospital Mölndal Sweden; ^4^ Department of Neurodegenerative Disease UCL Institute of Neurology London UK; ^5^ UK Dementia Research Institute at UCL London UK; ^6^ Hong Kong Centre for Neurodegenerative Diseases Hong Kong China; ^7^ Department of Clinical Science, Neurosciences Umeå University Umeå Sweden; ^8^ Department of Neurology Skåne University Hospital Lund Sweden; ^9^ Department of Clinical Sciences Lund University Lund Sweden; ^10^ Department of Neurology, Faculty of Medicine and Health Örebro University Örebro Sweden; ^11^ Department of Neurology and Department of Clinical and Experimental Medicine Linköping University Linköping Sweden; ^12^ Mathematical Sciences Chalmers University of Technology Gothenburg Sweden; ^13^ Department of Laboratory Medicine, Institute of Biomedicine, Sahlgrenska Academy University of Gothenburg Gothenburg Sweden

## Abstract

Axonal loss is the main cause of irreversible disability in multiple sclerosis (MS). Serum neurofilament light (sNfL) is a biomarker of axonal disintegration. In this nested case–control study, blood samples from 519 presymptomatic persons (age range 4–39 years) who later received an MS diagnosis showed higher sNfL concentrations than 519 matched controls (*p* < 0.0001), noticeable at least 10 years before clinical MS onset. Mean values for pre‐MS and control groups were 9.6 pg/mL versus 7.4 pg/mL 0–5 years before onset, and 6.4 pg/mL versus 5.8 pg/mL 5–10 years before onset. These results support that axonal injury occurs early in MS pathogenesis.

## Introduction

Axonal injury is a common feature of acute inflammatory demyelinating lesions in multiple sclerosis (MS) relapses,^1^ and chronic axonal loss is the main cause of irreversible disability in MS.^2^ Serum neurofilament light (sNfL) is a sensitive marker of acute and chronic axonal disintegration in neurodegenerative disorders^3^ and their presymptomatic stages.^4^ The sNfL level in MS is associated with relapses, EDSS progression, the presence of gadolinium‐enhancing MRI lesions, grey matter atrophy, and is predictive of long‐term outcomes.^5^, ^6^, ^7^ Cerebrospinal fluid NfL levels are reported to predict MS in individuals with radiologically isolated syndrome (RIS)^8^ and are associated with cognitive impairment.^9^

A study using the US Department of Defence Serum Repository reported elevated sNfL in samples collected a median of 6 (range 4–10) years before the onset of MS compared with controls, with a further increase at the time of onset.^10^ Confirmation is needed by larger studies with a more representative sex ratio, a wider age range and inclusion of other geographical regions.

We here examine neurofilament levels in deposited blood samples from persons who later received an MS diagnosis, by cross‐linking biobank samples with independently recorded data in the Swedish nationwide MS register. We investigate the onset of the presymptomatic axonal disintegration process.

## Patients and Methods

### Participants

In this nested case–control study, we used presymptomatically collected blood samples from 519 individuals who later received a diagnosis of relapsing–remitting MS (RRMS), and 519 matched controls.^11^, ^12^ Persons with RRMS were identified from the web‐based Swedish MS Register,^13^ established in 2002, and from a local MS database in Umeå. Data were exported in 2012, when the MS register contained 11,146 patients (www.neuroreg.se), and the Umeå database 2887, and were crosslinked with six Swedish biobanks. These biobanks contain serum samples stored after microbiological analyses performed at the University Hospitals of Skåne, Gothenburg, Örebro, Linköping and Umeå, as well as the Public Health Agency of Sweden. Controls were matched for biobank, sex, date of blood sampling, and date of birth in decreasing priority.

The study population had a median sampling age of 25 years (range 4–39 years), 82% were female, and the median time from sampling until disease onset was 9 years (range 1–32 years, Table 1). The time for first clinical symptoms of MS was extracted from the Swedish national MS registry and medical records. The register provides records on clinical variables including date of onset. Onset of MS is defined as the first symptom suggestive of a demyelinating event, which may be ascertained retrospectively.

**Table 1:** Characteristics of cases and controls.

Time from sampling until MS onset	ALL, *n* = 519	<5 years, *n* = 112	5–10 years, *n* = 163	10–15 years, *n* = 137	15–32 years, *n* = 107	10–32 years, *n* = 244
Sex (F), %	82	82	85	77	85	81
Age at sampling, years	25 (21–29)	27 (23–32)	25 (20–29)	25 (21–29)	22 (19–26)	24 (20–28)
Age at MS onset, years	35 (29–41)	30 (26–34)	32 (28–37)	37 (32–42)	43 (38–46)	40 (34–44)
Time from sampling until MS onset, years	9 (6–14)	2.9 (1.5–4.1)	7.4 (6.2–8.8)	12.1 (10.9–13.6)	19.1 (17.1–21.7)	14.4 (11.7–18.7)

Values expressed as a percentage for proportions and median (interquartile range) for continuous variables. MS, multiple sclerosis; F, female.

The study was approved by the research ethics board of Umeå Sweden 2011–198/31 (2011‐08‐03), and Addendum 2019–03402 (2019‐08‐19). Participants were informed with an opt‐out option.

### Laboratory method

sNfL concentration was measured using Single molecule array (Simoa) technology and the NF‐Light assay on an HD‐X Analyser according to instructions from the kit manufacturer (Quanterix, Billerica, MA). All samples were measured in one round of experiments using one batch of reagents by board‐certified laboratory technicians blinded to clinical data. For a QC sample with an NfL concentration of 6.8 pg/mL, repeatability was 11.2% and intermediate precision was 11.2%. For a QC sample with a concentration of 50.3 pg/mL, repeatability was 6.9% and intermediate precision was 10.6%.

### Statistical methods

Individual log ratios of sNfL (pre‐MS/matched controls) were plotted against time to MS onset and the relationship estimated with smoothed regression analysis using the loess function in R. Serum NfL concentrations were log‐transformed, and paired *t*‐tests were used to compare cases with their matched controls in the whole sample as well as in 5‐year time groups until MS onset.

Conditional logistic regression with MS as outcome and log sNfL as predictor were used to estimate the odds ratio (OR) for the total group and 5‐year time groups until MS onset. Since sNfL is analysed on a log scale, the ORs are for a 10‐fold increase in sNfL.

Individual log ratios of sNfL were further analysed with linear regression using time to onset as well as age at sampling as predictors in the matched pairs where the time to onset was <10 years.

## Results

**Figure 1:**
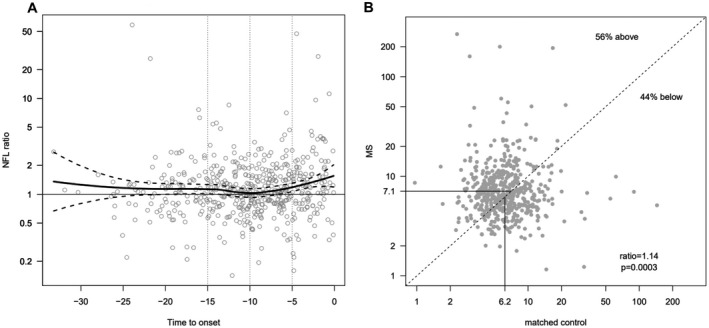
(A) Scatterplot with locally estimated smoothing curves for sNfL against time to MS onset. Pre‐MS/matched controls ratios for sNfL against time until MS onset. After a vague initial phase, the ratio shows a steadier increase from approximately 10 years before MS onset. (B) Paired *t*‐test of sNfL for pre‐MS cases vs. matched controls. Case control ratios for sNfL, showing 56% of ratios above the identity line and geometric mean values for MS of 7.1 compared with 6.2 for controls. sNfL, serum neurofilament light; MS, multiple sclerosis.

Loess regression of log sNfL for ratios between individual pre‐MS and matched control values as a function of time to MS onset showed an increasing trend from at least 10 years before MS onset (Fig. 1A). Serum NfL was significantly higher in the total pre‐MS group, with a geometric mean level of 7.1 pg/mL (CI 6.8–7.4) compared with 6.2 pg/mL (CI 5.9–6.5) in the control group (*p* = 0.00001, Fig. 1B). The magnitude of this difference increased closer to the onset of symptoms.

**Figure 2:**
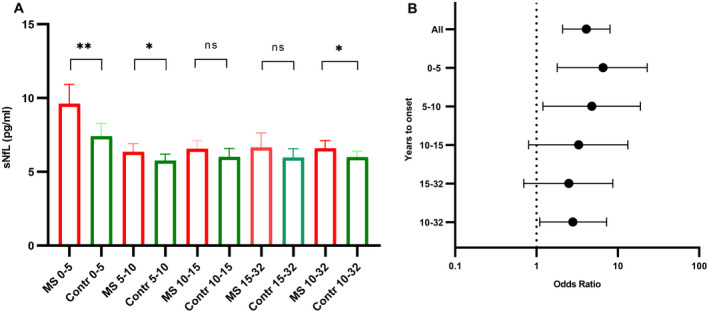
(A) Geometric mean with significance level estimated from paired *t*‐tests for different times until MS onset. Error bars show 95% CI. (B) Odds ratio for a 10‐fold increase in sNfL regarding different times until MS onset. MS, multiple sclerosis; sNfL, serum neurofilament light. *p ≤ 0.05; **p ≤ 0.05. [Colour figure can be viewed at wileyonlinelibrary.com]

sNFL levels showed significant differences 0–5 years before onset, with a geometric mean value of 9.6 pg/mL (CI 8.4–10.9) in the pre‐MS group, compared with 7.4 pg/mL (CI 6.7–8.3) in controls (*p* = 0.002), and 5–10 years before onset with a pre‐MS mean of 6.4 pg/mL (CI 5.9–7.0) and a mean of 5.8 (CI 5.4–6.2) in controls (*p* = 0.02). There was no significant difference for the 10–15‐year and 15–32‐year groups; however, when merged (10–32 years prior to onset of symptoms) a significant difference (*p* = 0.026) was seen (Fig. 2A).

A 10‐fold increase in sNfL levels was associated with an increased risk of MS (OR 4.1, CI 2.1–8.0). For the 0–5‐year group, pre‐MS OR was 6.6 (CI 1.8–23). For the 5–10‐year group, pre‐MS OR was 4.8 (CI 1.2–19). No significance was found for 10–14 years (OR 3.3, CI 0.8–13.3) or above 15 years (OR 2.5, CI 0.7–8.7). However, increasing the samples by merging the latter groups, that is, 10 years and above, shows a significant increased risk of MS (OR 2.8, CI 1.1–7.3) (Fig. 2B).

A regression analysis of the log sNfL ratio on time to onset based on matched sets with time to onset <10 years gives a yearly increase of 4.5% of the sNfL ratio (*p* = 0.004), while regression on age at sampling for the same data is not significant (*p* = 0.97).

## Discussion

We found an increase in sNfL that starts at least 10 years before relapsing remitting MS onset. These data strongly suggest that neuroaxonal injury gradually starts in some individuals at least 10 years before the focal neurological onset of MS, although the 10‐year limit was not distinct. Individuals in the pre‐MS group are sampled at widely different time spans from the onset of clinical MS, and many pre‐MS individuals had a normal sNfL value. We observed a difference in sNfL compared with matched controls for individuals with values in the low, intermediate and high range. Likely, multiple inflammatory demyelinating events produce a cumulative effect on sNfL, with heterogeneity between individuals concerning number, severity of lesions and location. Chronic active lesions are known to occur in RRMS and RIS,^14^ and axonal degeneration tends to start gradually around these chronical active lesions.^15^

In the national register, MS onset is defined by the first episode suggestive of a demyelinating attack in an individual later determined to have definite MS. However, an increasing number of unspecific events or symptoms have been reported recently as constituting a clinical prodrome present at least 5 years before onset. This prodrome may include depression, fatigue, sleep disorders, pain and bladder issues and even non‐neurological symptoms such as anaemia.^16^, ^17^, ^18^ One group has proposed that such prodromes are early MS symptoms.^19^ We observed an incipient increase of sNfL at least 10 years before the onset of MS, probably often encompassing the prodrome.

The difference in sNfL between the pre‐MS and control groups increased closer to onset. Limiting the observation time to 10 years before onset, the pre‐MS/control sNfL ratio was significantly associated with the time to onset, whereas we did not observe any association between the pre‐MS/control sNfL ratio and the age at sampling. Therefore, factors initiating the axonal disintegration are mainly associated with the ongoing or imminent onset of MS pathology, conceivably inflammation, rather than the age of the individual.

Our observation is based on ages 4–39 at sampling and a range of 1–32 years from sampling until disease covering a broader range of age and duration until onset than in a previous study.^10^

The Swedish microbiological biobanks used in this study comprise specimens for either diagnostic or screening purposes. Earlier studies have shown that sNfL is elevated in several infections.^20^ This random factor affecting both our pre‐MS and control materials did not conceal the time dependent increase in the pre‐MS/control ratio.

The substantial overlap in sNfL between pre‐MS and control groups in the present study prevents useful prediction in a population‐based screening, however increased sNfL levels may constitute a convenient search criterion combined with other predictive factors such as RIS or heredity. Especially if an increase in sNfL was detected in consecutive samples from the same individual. The lack of longitudinal samples is a limitation of the current study. Further studies are needed to search for a possible dominant order of appearance of prodromal MS symptoms and the longitudinal sNfL response. The present study provides an indication of the duration of a presymptomatic axonaldegenerative phase in MS.

## Author Contributions

D. J., P. S. and O. A. contributed to the conception and design of the study; all authors contributed to the acquisition and analysis of data; D. J., S. N., P. S. and O. A. contributed to drafting the text and preparing the figures.

## Conflict of Interest

Nothing to report.
